# Asymptotic freedom and noninteger dimensionality

**DOI:** 10.1038/s41598-021-83002-9

**Published:** 2021-02-09

**Authors:** Subhash Kak

**Affiliations:** Oklahoma State University, Stillwater, Stillwater, USA

**Keywords:** Mathematics and computing, Information theory and computation

## Abstract

This paper shows that below a critical value of dimensionality that lies between two and three, the potential between objects begins to fall as the energy levels increase. For dimensionality below two, the potential becomes constant irrespective of separation and the force between them disappears, which represents a new paradigm of asymptotic freedom. Since asymptotic freedom is at the basis of many applications such as those associated with strange metals, unconventional superconductors, and fractional quantum Hall states, the new paradigm can have novel applications. It also is of relevance to the study of anomalous mechanical effects that are important in metamaterials.

## Introduction

It was recently shown^1^ that taking the physical space to have noninteger dimensionality explains the origins of the inverse square law and resolves the discrepancy in two different estimates of the Hubble constant. In this paper, we investigate a further implication of this theory to the anomalous situation of strong interaction at large distances and much weaker interaction at short distances, which is a characteristic of asymptotic freedom^2–4^.

**Figure 1:**
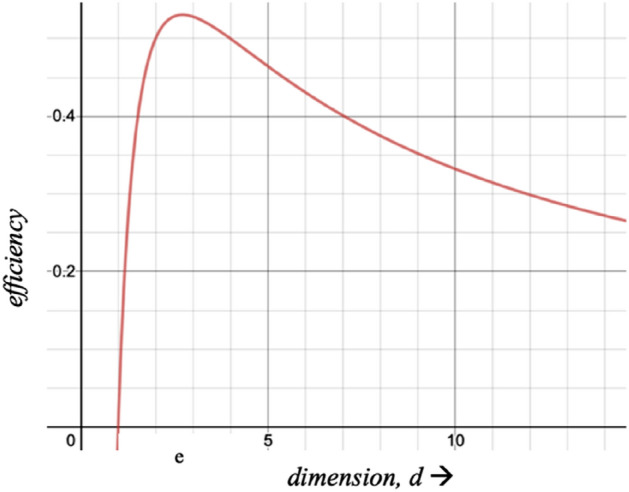
Dimensional efficiency is maximum at d = e.

From the perspective of information theory, the optimum number of dimensions is the noninteger (and irrational) number *e* and since Nature chooses optimality, this should be the dimensionality of physical space^1,5^ and the inverse square law itself may be seen as a consequence of noninteger dimensionality^6^. The information efficiency per dimension is $E\left( d \right) = \frac{\ln d}{d}$ (Fig. 1 shows the efficiency rise from zero for *d* = 1, with peak at *e*) and the intrinsic dimensionality of data is also *e*^7,8^. As dimensionality and information are foundational to the understanding of physical reality, these ideas ought to be of relevance in the understanding of asymptotic freedom.

Asymptotic freedom is counterintuitive^9^, and it plays a role in novel states of matter that include strange metals, unconventional superconductors^10,11^, and fractional quantum Hall states^12–14^. As a property of some gauge theories, it offers a mechanism for confinement at large distance by invoking properties of the mathematical structures used to describe these interactions.

Other counterintuitive phenomena are associated with negative differential response, and they are to be found not only in physics but also in chemistry and biological processes^15^. In materials that exhibit negative linear compressibility, there is expansion in one or more directions during the process of uniform compression^16–18^ and this anomalous mechanical behavior has found applications in the design of pressure sensors, artificial muscles and actuators^19–21^.

This counterintuitive nature is sometimes understood through the lens of the complementarity principle^22^, which provides a way to view seemingly inconsistent descriptions as two sides of the same reality. Although, the principle arose originally in quantum theory, it has been argued that it should apply much more broadly to diverse fields of science^23^, including biological phenomena^24^. Complementarity is the description of the same phenomenon in distinct, categorically different ways that cannot be done in the same spatial, temporal, or situational context.

The notion of dimension applies to physical reality at all conceivable scales, therefore one must also consider anomalous mechanical properties of materials that emerge from compressing three-dimensional volumes into lower dimensions^25–27^, which may be seen as an example of increasing the energy scale, with prospective applications to superconductivity, ferroelectricity, information communication, sensing and detection^28–30^.

Some of the inconsistencies in the interpretation of phenomena could be the consequence of different ways space is considered in the theory. As stressed by Landauer^31^, information is physical and, therefore, information-theoretic ideas must play a key role in the understanding of the above-mentioned anomalous phenomena.

In this paper, rather than examining the application of noninteger dimensionality to any of the above phenomena directly, we show that a novel version of asymptotic freedom is associated with noninteger dimensionality. This represents an unexpected new framework with potential applications similar to those of anomalous mechanical properties of materials. Also, since gauge transformations relate to the very nature of space, dimensionality may be of relevance to gauge theories.

## Dependence on *d*

We can visualize a given noninteger space sitting within the container of the ceiling integer space (e.g. 1.7-dimensional space sits within the 2-dimensional space). It may be argued that a fundamental characteristic of a noninteger space is that of a continual shrinking of the metrical relationships between objects^1,6^. This is seen most clearly when we visualize a 2-dimensional space obtained from a 3-dimensional space which will cause all the points in the third dimension to collapse to the extant two dimensions.

Consider the example of a noninteger space with dimension *d*, which we shall compare with its ceiling function *d* the smallest integer greater than it. Since dimensionality is an additive property, we can distribute the fractional part along any direction. Thus *d* = 1.8 (within the implicit 2-dimensional ceiling space) would imply a dimension of 0.9 along the one-dimensional infinite line in any direction.

The view of the noninteger dimension that there are gaps in the space is complementary to the view that relative measures on the line *tend to shrink* in proportion to the value of dimensionality, and the tendency to do so is a function of the ratio $\frac{{\left( {d - d} \right)}}{d} = \frac{1}{d}f\left( {d - d} \right),$ where *d* is the ceiling function of *d,* and $f\left( {d - d} \right)$ represents the functional relationship with respect to the departure of the dimension value from its ceiling integer. For the example of *d* = 1.8, the shrinking is a function of $\frac{1}{2}f\left( {0.2} \right)$.

The tendency for space to contract constitutes a potential, making points associated with events that the observer witnesses to tend to come closer to each other. This potential leads to dynamics that emerge thus from the very nature of space. This potential is not assumed to have an a priori existence, independent of dimensions, and therefore it represents a view different from the current understanding.

Since *d* characterizes the space, the potential between two objects with unit measures must also be inversely related to the separation so that objects that are further apart have less influence on each other than those that are near. Points on a 1-dimensional infinite space stay where they are, whereas those on a 0.8-dimensional infinite space will have a potential to come closer by the 0.8 measure. Consider two objects with unit measures with a separation of *r*, and let the potential be defined by $p_{d} \left( {d,r} \right)$.

The sphere representing the equipotential surfaces at a distance of *r* has surface equal to $S_{d} \left( {d,r} \right),$ but the distance *r* should be modified to $\frac{d r}{d}$ because the density of the space along any line is smaller by the fraction $\frac{d}{d}.$ Therefore, the potential will be proportional to

1 $$p_{d} \left( {d,r} \right) = \frac{{f\left( {d - d} \right) d r}}{{d^{2} }} \times \frac{1}{{S_{d} \left( {d,r} \right){ }}} = \frac{{f\left( {d - d} \right) d r}}{{d^{2} S_{d} \left( {d,r} \right)}}$$

The value of *S*_*d*_ for 3- and 2- and 1-dimensional worlds is 4*π*r^2^, 2*πr*, and *0*, respectively, and these are the surface area of a sphere, the circumference of a circle, and the length of a point. Corrected for reduced density, the expression for $S_{3} \left( {d,r} \right),$ and $S_{2} \left( {d,r} \right)$ becomes:

2 $$S_{3} \left( {d,r} \right) = \frac{4}{9}\pi r^{2} d^{2} ; \quad S_{2} \left( {d,r} \right) = \frac{2\pi rd}{2} = \pi rd$$

The potentials for the ranges 2 < *d* < 3 and 1 < *d* < 2, that is $p_{3} \left( {d,r} \right)$ and $p_{2} \left( {d,r} \right),$ will be:

3 $$p_{3} \left( {d,r} \right) = \frac{{f\left( {3 - d} \right)}}{4\pi rd};\quad p_{2} \left( {d,r} \right) = \frac{{f\left( {2 - d} \right)}}{4\pi }$$

Note that $p_{2} \left( {d,r} \right)$ is independent of *r*.

The forces corresponding to these potentials will be the derivative of the expressions (3). For a fixed *d*, over the range 2 < *d* < 3, the potential is proportional to 1/*r* and therefore the forces between objects in such a space will be proportional to $${\raise0.7ex\hbox{$1$} \!\mathord{\left/ {\vphantom {1 {r^{2} }}}\right.\kern-\nulldelimiterspace} \!\lower0.7ex\hbox{${r^{2} }$}}$$. This is the origin of the inverse square law^1,6^.

The potentials as functions of dimensionality alone (in which case we simply drop *r* as a variable) are:

4 $$p_{3} \left( d \right) = \frac{{f\left( {3 - d} \right)}}{4\pi d};\quad p_{2} \left( d \right) = \frac{{f\left( {2 - d} \right)}}{4\pi }$$

We need a theoretical framework to find these functions. In what follows, we use mathematical constraints to propose some candidates for these functions.

## The function *p*_3_(*d*)

Some further constraints are needed to find plausible candidates for $f\left( {3 - d} \right).$ This function should be zero for the values just outside the interval 2 < *d* < 3 at *d* = 2 and 3. The functions should be universal for unconstrained systems (as in cosmology), but would depend on external constraints for an engineered system.

*A matched distribution.* If physical structures are compared to the optimal filter corresponding to natural distribution, then the use of optimum signal theory^32^ will require that the potentials be matched to the efficiencies associated with the *d*-values.

One can associate a pdf with the dimension *d* and call it the DED (dimensional efficiency distribution) *f*(*d*) with the range from 1 to the maximum of *N*:

5 $$f\left( d \right) = \frac{2}{{(\ln N)^{2} }}\frac{\ln d}{d},\quad 1 \le d \le N$$

where the factor $\frac{2}{{(\ln N)^{2} }}$ is to ensure that the area under it is 1. Its expectation is $E\left( D \right) = \frac{2}{{(\ln N)^{2} }} (N\ln N - N + 1)$, and its variance is

$$VAR\left( D \right) = \frac{2}{{(\ln N)^{2} }}\left( {\frac{\ln N}{2} N^{2} - \frac{{N^{2} }}{4} + \frac{1}{4}} \right) - E\left( D \right)^{2} .$$

The value of *N* that is of interest to those looking at physical dimensions is 3. For such a case $\frac{2}{{(\ln N)^{2} }} \cong 1.657$ and $E\left( D \right) \cong 2.147$ and $VAR\left( D \right) \cong 0.268.$ Although the optimal value of dimensionality is *e*, the expected value of dimension is substantially less at 2.148 with a variance of 0.268.

If *N* = *e,* $E\left( D \right) = 2$ and $VAR\left( D \right) \cong 0.195.$ One can define a generalization of the DED as follows: $f_{gen} \left( {a, d} \right) = \frac{2a}{{({\text{ln a}}N)^{2} }}\frac{\ln ad}{d}, 1 \le d \le N$, with the discrete version $p\left( n \right) = \frac{1}{K}\frac{\ln an}{n}, 1 \le n \le N$ where $K = \sum\nolimits_{i = 1}^{N} {\ln \left( {ia} \right)^{1/i} }$.

**Figure 2:**
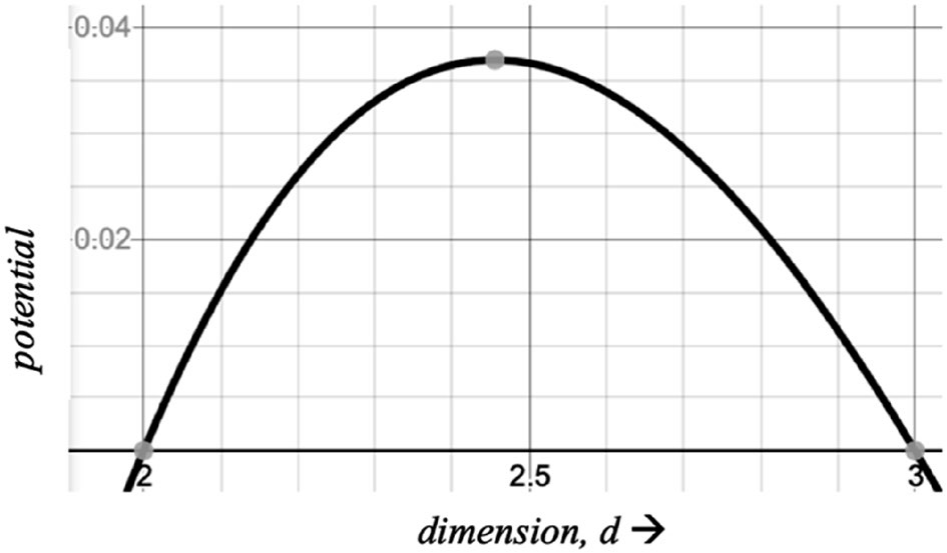
Potential $p_{3,A} \left( d \right)$ with peak at 2.455.

To return to the *d*-dimensional space acting as a matched filter, one may choose the following function:

$$f_{A} \left( {3 - d} \right) = \left( {d - 2} \right)\left( {3 - d} \right)\frac{\ln d}{d}$$

with $p_{3,A} \left( d \right) \propto \left( {d - 2} \right)\left( {3 - d} \right)$ $\frac{lnd}{{d^{2} }}$, which is shown in Fig. 2. It has a peak at *d* = 2.455.

Now we consider two ad hoc functions that satisfy the constraints of zero values at 2 and 3. The first of these is the same as that of Fig. 2 excepting for the normalizing weight, which is linear with respect to these constraints; the second is linear with respect to 2 and exponential with respect to 3 with exponent of 0.25.

6 $$f_{B} \left( {3 - d} \right) = \left( {d - 2} \right)\left( {3 - d} \right)$$

7 $$f_{C} \left( {3 - d} \right) = \left( {d - 2} \right)\left( {e^{{\left( {3 - d} \right)}} - 1} \right)^{1/4}$$

The potential function will then have the following characteristics:

8 $$p_{3,B} \left( d \right) = \frac{{\left( {d - 2} \right)\left( {3 - d} \right)}}{4\pi d}$$

9 $$p_{3,C} \left( d \right) = \frac{{\left( {d - 2} \right)\left( {e^{{\left( {3 - d} \right)}} - 1} \right)^{1/4} }}{4\pi d}$$

## The critical dimension *d*_*crit*_

**Figure 3:**
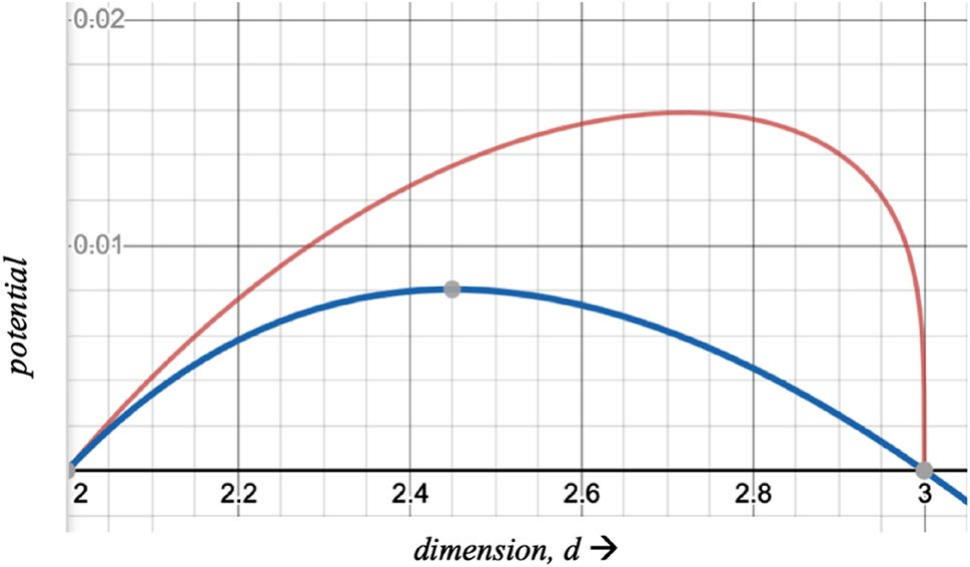
Potential with respect to dimensionality; blue line $p_{3,B} \left( d \right),$ and red line $p_{3,C} \left( d \right)$.

Figure 3 is a map of the above potential functions. The peak for the blue curve (Eq. 8) is at *d* = 2.4495, whereas the peak for the red curve (Eq. 9) is at *d* = 2.7195. This latter maximum is quite close to the optimal value of *e*. The value of the dimensionality at which the potential is maximum will be termed *d*_*crit*_ the critical dimension.

The continuing reduction of dimensionality is further injection of energy into the system. Figures 2 and 3 show that as we move from the right to the left with decreasing value of *d* until *d*_*crit*_ is reached, we witness increasing energy density in the space and the potential increases, which means that the interaction amongst the particles increases. But beyond *d*_*crit*_, which in the three cases of these figures occurs somewhere between 2 and 3, we come across *counterintuitive behavior* where increasing energy (equivalent to reducing dimensionality) reduces the strength of interaction. Increasing dimensionality beyond the critical dimension also reduces this strength, and this behavior has obvious applications for understanding evolution in cosmology.

## Asymptotic freedom for *1* < *d* < *2*

For situations where the ceiling dimensionality is 2, the potential function $p_{2} \left( {d,r} \right) = \frac{{f\left( {2 - d} \right)}}{4\pi }$ being independent of *r* implies that objects in such a space will experience the same potential and, therefore, will behave *as if in a bag,* and experience no force that varies with the separation. There will be a dependence on *d*, that can be put to use in engineering applications.

Since there is no dependence with respect to separation, this represents a case of *asymptotic freedom* that is arrived at by squeezing the dimensionality of space. This fact represents a most unexpected property associated with the dimensionality of noninteger spaces.

Although there is no noninteger space generated intrinsic dynamics for the case of *1* < *d* < *2,* there could be dynamics as a consequence of externally applied forces that need to be analyzed separately.

## Conclusions

This paper explored properties of noninteger dimensionality and examined how interaction potentials across objects vary. It was shown that as the dimensionality falls below *d*_*crit*_ somewhere around the mid-point of 2 and 3, the potential begins to fall, signifying a reduction in the interactions amongst the particles. At *d* = 2 and below, it becomes constant, irrespective of separation, and the force between objects disappears, which represents a case of asymptotic freedom that can be the basis of novel applications. This is a surprising property of noninteger spaces.

We have shown that objects in a 2-dimensional space will not interact with each other, but as the dimensionality increases beyond 2, the interactions begin to increase in strength until the point of maximum strength. Systems could be engineered where the difference in these regimes is exploited to perform specific signal processing or anomalous mechanical function. This also opens up the question of how to control the value of the critical dimension that is likely to be an important variable in the design of metamaterials.

The work in this paper provides a dimensionality-based explanation of interactions becoming weaker as energy scales increase. Whether this phenomenon based on dimensionality of space has any connection with other models of asymptotic freedom remains to be investigated.
